# *Lactobacillus* strains vary in their ability to interact with human endometrial stromal cells

**DOI:** 10.1371/journal.pone.0238993

**Published:** 2020-09-14

**Authors:** Megan Shiroda, Shannon D. Manning

**Affiliations:** Department of Microbiology and Molecular Genetics, Michigan State University, East Lansing, MI, United States of America; University of Western Australia, AUSTRALIA

## Abstract

The placental membranes that surround the fetus during pregnancy were suggested to contain a low abundance microbiota. Specifically, abundance of *Lactobacillus*, a probiotic and dominant member of the microbiome of the lower reproductive tract, has been shown to correlate with healthy, term pregnancies. We therefore sought to assess the interactions between four different *Lactobacillus* strains with immortalized decidualized endometrial cells (dT-HESCs), which were used as a model to represent the outermost layer of the placental membranes. Notably, we demonstrated that all four *Lactobacillus* strains could associate with dT-HESCs *in vitro*. *L*. *crispatus* was significantly more successful (p < 0.00005), with 10.6% of bacteria attaching to the host cells compared to an average of 0.8% for the remaining three strains. The four strains also varied in their ability to form biofilms. Dependent on media type, *L*. *reuteri* 6475 formed the strongest biofilms *in vitro*. To examine the impact on immune responses, levels of total and phosphorylated protein p38, a member of the Mitogen Activated Protein Kinase (MAPK) pathway, were examined following *Lactobacillus* association with dT-HESCs. Total levels of p38 were reduced to an average of 44% that of the cells without *Lactobacillus* (p < 0.05). While a trend towards a reduction in phosphorylated p38 was observed, this difference was not significant (p > 0.05). In addition, association with *Lactobacillus* did not result in increased host cell death. Collectively, these data suggest that varying types of *Lactobacillus* can attach to the outermost cells of the placental membranes and that these interactions do not contribute to inflammatory responses or host cell death. To our knowledge this is the first *in vitro* study to support the ability of *Lactobacillus* to interact with placental cells, which is important when considering its use as a potential probiotic within the reproductive tract.

## Introduction

The placental membranes surround the fetus during pregnancy and are made of two layers, the amnion and choriodecidua, held together by connective tissue and fibroblast cells. These layers serve as a protective barrier between the fetus and ascending pathogens from the vaginal tract. Infection of these membranes is thought to cause weakening of the membranes, leading to miscarriage, preterm birth or neonatal sepsis [1, 2]. While previously considered sterile, some metagenomic studies have found that the placenta and placental membranes may have a small load of commensal bacteria including *Acinetobacter*, *Escherichia*, *Enterobacter* and *Lactobacillus* [3, 4]. Some bacteria, including members of *Lactobacillus*, have been detected in healthy, term pregnancies [4] and in placental samples from low gestational age neonates [5]. Importantly, no tissue culture studies have been performed to determine whether *Lactobacillus* can interact with the host cells in the placental membranes *in vitro*.

Many lactobacilli exist as commensals dominating the healthy vaginal tract of women [6]. The positive impact of key *Lactobacillus* spp. on human health has been studied in the gut and urogenital tract, but their effects in the upper reproductive tract or on the placental membranes have not been fully examined. In addition, the source of lactobacilli populations residing in these regions is not clear. Nonetheless, commensal microbes, including some *Lactobacillus spp*., can modulate host immune responses and directly or indirectly inhibit colonization and invasion by pathogens [7]. Indirect inhibition occurs when host attachment sites are occupied by commensals that compete for nutrients and growth factors, produce unique metabolites to enhance survival, and stimulate the production of factors with pro-inflammatory and antimicrobial effects [8, 9]. Such indirect inhibition is often referred to as colonization resistance. Direct inhibition can include production of lactic acid or other secondary metabolites. In the urogenital tract, lactobacilli have been found to inhibit a variety of individual pathogens including human immunodeficiency virus (HIV) [10, 11], uropathogenic *Escherichia coli* (UPEC) [12, 13], Group B *Streptococcus* (GBS) [14–16], *Neisseria gonorrhoeae*, and *Gardnerella vaginalis* [17, 18]. Some *Lactobacillus* spp. have also been shown to counteract more complex disease states such as bacterial vaginosis [19].

Immune modulation by lactobacilli can be localized or systematic. Mechanisms include increased production of immunoglobulins (IgA), modulation of cytokine production, inhibition of the Mitogen-activated protein kinase (MAPK) signaling pathways, and increased phagocytosis by immune cells [20, 21]. Modulation of the immune system has been more heavily studied in the gut, but recent studies suggest that the presence of some *Lactobacillus* spp. may also play a role in the urogenital tract. Supernatants from lactobacilli, for example, were shown to affect cytokine responses to lipopolysaccharide (LPS) in the decidua, reducing a potentially harmful inflammatory response during pregnancy [22], while another study found no evidence of inflammation in the placental villous space despite the presence of microbes by 16S *in situ* hybridization [23]. As most *Lactobacillus* spp. are considered to be safe, their efficacy as a probiotic in the urogenital tract during pregnancy is still under investigation. Most prior studies have primarily focused on examining the ability of individual strains or species of lactobacillus to reduce pathogen colonization in the vaginal tract [15, 24, 25].

To better understand the interactions between live lactobacilli and the placental membranes, we sought to characterize the interactions of four different *Lactobacillus* strains with a cell line model representing the outermost layer of cells of the placental membranes. Using this model, we assessed whether these lactobacilli maintain a beneficial interaction with these cells to provide similar protective functions as has been described in other body sites. Due to known variation between *Lactobacillus* spp. and strains, we also characterized growth and biofilm phenotypes of each strain.

## Materials and methods

### Bacterial strains and growth conditions

*Lactobacillus* strains were selected to represent species that have been found in both the vagina and extraplacental membranes in prior microbiome studies [4, 26]. Because previous work has focused on certain beneficial species such as *L*. *reuteri* (Lr), *L*. *gasseri* (Lg) and *L*. *crispatus* (Lc), these species were selected for use. Lr6475 was isolated from breast milk (MM4-1A PTA-6475), Lg33323 from the vagina (DSM 20243 [63 AM]), and Lc19390 from stool. One additional *L*. *reuteri* strain, Lr17938, was also selected. This Lr17938 strain is the daughter strain of *L*. *reuteri* ATCC 55730, which was isolated from breast milk and was shown to possess transferable resistance traits for tetracycline and lincomycin [27]. Lr17938, however, no longer carries this resistance plasmid.

All four *Lactobacillus* strains were cultured in de Man, Rogosa and Sharpe (MRS) broth (Difco 288130), a standard growth broth for *Lactobacillus*, or MRS agar at 37°C with 5% CO_2_. *Lactobacillus* growth was evaluated by diluting an overnight culture of *Lactobacillus* 1:10 and measuring growth at an optical density (OD) of 595 for 8 hours in a BioTek Cytation 3 Imager. Two GBS strains, GB00411 and GB01048, were selected to represent a weak and strong biofilm control [28], respectively, for the *Lactobacillus* biofilm assays. Both GBS strains were grown overnight in Todd Hewitt broth at 37°C with 5% CO_2_. For the subsequent experiments, starting inoculums were prepared by taking the equivalent of 0.1 OD_595_ of an overnight culture, washing twice with 1x phosphate buffered saline (PBS). The final inoculum was prepared in the appropriate media for the given experiment. Equal colony forming units (CFUs) (10^7^) of each bacterium by OD_595_ were confirmed by plate counts (data not shown).

### Biofilm production

Overnight cultures of *Lactobacillus* were inoculated into fresh Tryptic Soy Broth (TSB) + 1% dextrose (TSBd) or MRS in a 96 well plate at an OD_595_ of 0.1. Each biological replicate contained at least four technical replicates and media controls. Plates were incubated for 48 hours and subsequently washed twice with 1x PBS to removal non-adherent cells. 100μl of 3% crystal violet was added to each well and incubated for 10 minutes. Wells were washed four times with 1x PBS, and 200μl of ethanol was added to solubilize the crystal violet. A total of 50μl was added to a fresh plate for an absorbance reading. To quantify, the media control numbers were subtracted and each resulting number was multiplied by four. Technical replicates were averaged within a plate and at least three biological replicates were performed. Significance was determined using an unpaired analysis of variance (ANOVA) after confirmation of a normal distribution by the Shapiro-Wilk test.

For comparison, two GBS strains, GB01048 and GB00411, were examined. These strains were previously classified as strong and weak biofilm formers using the same biofilm quantification method [28], while GB00411 was found to be cytotoxic to dT-HESCs [29]. *Lactobacillus* strains with a final OD_595_ falling between these two GBS strains (1.13–7.00 OD_595_) were considered high biofilm formers. Those with values less than GB00411, however, were considered weak biofilm formers (1.13–0.25 OD_595_). To classify a strain as a non-biofilm former, a previously described method was used [30]. Briefly, an OD_595_ cut-off value (ODc) was assigned based on the internal negative control and its standard deviations across three biological replicates (ODc = negative control + 3 standard deviations = 0.25 OD). Non-biofilm producers fell below the ODc.

### Cell culture

Telomerase-immortalized human endometrial stromal cells (T-HESC, ATCC CRL-4003) [31] were cultured in DMEM/Nutrient Mixture Ham's F-12 with l-glutamine (Sigma Aldrich, St. Louis, MO), 1% BD ITS+ Universal Culture Supplement Premix (BD Biosciences, San Jose, CA), 1.5 g/liter sodium bicarbonate, 2% penicillin / streptomycin, and 10% charcoal-treated FBS (HyClone), which is referred to as HESC medium herein. For the cell experiments, T-HESCs were decidualized (dT-HESCs) as described [19, 20] by incubating the cells with 0.5 mM 8-bromo-cyclic amp (cAMP) (Sigma Aldrich) for three to six days. Completely confluent monolayers were used for all experiments to ensure that only decidual cell surfaces were available for bacterial interactions.

### Association of *Lactobacillus* strains with decidual cells

Association assays were performed as described previously [28, 32]. Briefly, monolayers of decidual cells were washed thrice with 1x PBS before infection with bacterial cultures. Overnight cultures of *Lactobacillus* were washed once with PBS and re-suspended in infection media (T-HESC medium without ITS+ or antibiotics and with 2% charcoal-treated FBS). dT-HESC cells were infected at a multiplicity of infection (MOI) of ten lactobacilli per host cell (MOI = 10), as confirmed by both bacterial (CFUs) and HESC counts. Following a two-hour incubation at 37ᵒC in atmospheric conditions, samples were taken from each well to determine the final CFUs of *Lactobacillus*. To quantify the number of associated cells, wells were washed three times to remove unattached cells. Host cells were disrupted using Triton-X, and wells were scraped thoroughly. Repeated pipetting and vortexing ensured even resuspension before plating. The percent of associated cells was calculated by dividing the CFU of associated cells by the final CFU of each well. These experiments were performed in three to four biological replicates of technical triplicates. Significance was determined using an unpaired ANOVA following a Shapiro-Wilk normalcy test. The ability of lactobacilli to survive in Triton-X was confirmed using the same protocol described but without dT-HESCs.

### Cytotoxicity assays

Monolayers of dT-HESCs were cultured in 24-well plates and cells were incubated with lactobacilli (MOI = 10) using a protocol for GBS described by Korir, et al. [32]. After incubation for four hours, cells were washed twice with 1x PBS, treated with 4μM ethidium homodimer 1 (Molecular Probes, Eugene, OR), suspended in 1x PBS, and incubated at room temperature for 30 minutes without light. Fluorescence was measured at 528-nm excitation and 617-nm emission using a plate reader (Beckman Coulter, Inc). As we have done previously [29], the total number of cells in each well was calculated by adding 0.1% (wt/v) Saponin (Sigma Aldrich) and incubating for at least 20 minutes before repeating the fluorescence reading. The percent permeability (cell death) was calculated by dividing the initial reading by the second and multiplying by 100. Comparisons were made to GB00411, which was found to be cytotoxic to dT-HESCs in our prior studies [29]. These experiments were performed in three to four biological replicates of technical triplicates and significance was determined using an unpaired ANOVA after confirmation of a normal distribution using the Shapiro-Wilk test.

### Detection of IL-10 by ELISA

dT-HESCs were incubated with *Lactobacillus* (MOI = 10) for three hours. Cell supernatants were collected, centrifuged at 2,400 rcf for 20 minutes to remove bacteria and cellular debris, and stored at -20°C. Concentration of IL-10 was determined using the IL-10 Human ELISA kit: ab100549 (Abcam, Cambridge, MA) according to manufacturer’s instructions; comparisons were made relative to mock infection. Data from three biological replicates were pooled to quantify the average cytokine concentrations (pg/mL). Significance was determined by unpaired ANOVA and post-hoc Dunnett’s tests relative to mock infection.

### Western blotting for p38

dT-HESC were incubated with *Lactobacillus* (MOI = 10) and protein lysates were collected following a three-hour infection to quantify the abundance of p38 as described [29]. Briefly, cells were washed twice with 1x PBS and lysed with 300μl of Kinexus lysis buffer (Kinexus, Vancouver, Canada) for ten minutes at 4°C. Each well was scraped, and the lysate was collected. Samples were spun at 2400 rcf for 20 minutes to pellet cellular debris, transferred to a fresh tube, and stored at -20°C. Protein concentration was determined by bicinchoninic acid (BCA) assay (Pierce) using Bovine Serum Albumin (BSA) immediately prior to separation on a 4–15% polyacrylamide gel (BioRad). Samples were transferred to a polyvinylidene difluoride (PVDF) membrane and the membrane was blocked in 5% BSA (Fisher Scientific) + 0.1% Tween 20 (Sigma) in 1x Tris-Buffered Saline (TBS) for at least two hours.

The membranes were exposed to primary rabbit monoclonal antibodies (1:1000) for phospho MAPK-p38 (T180+Y182; #4511S, Cell Signaling Technology, raised using synthetic phosphopeptide corresponding to residues surrounding Thr180/Tyr182 of human p38 MAPK), total MAPK p38 (1:1000; #8690S, Cell Signaling, raised using synthetic peptide corresponding to residues near the carboxy terminus of human p38 protein), or beta-tubulin (1:500; #2128S, Cell Signaling Technology, raised using synthetic peptide corresponding to the amino terminus of human β-tubulin) overnight at 4°C as previously described [23]. Membranes were washed five times over an hour in 1x TBS+0.1% Tween 20 and incubated with goat-anti-rabbit IgG-HRP secondary antibodies (1:5000, Life Technologies) at room temperature for 1.5 hours followed by another set of wash steps in 1x TBS+0.1% Tween 20. The membranes were incubated with ECL chemiluminescence reagent (Pierce) prior to developing using an Amersham Imager 600 (GE Life Sciences). Relative abundance of the proteins was determined using Image J from three independent replicates. Beta tubulin levels per well were used for normalizing to protein levels to control for loading differences. Significance was determined with an unpaired ANOVA and post-hoc Dunnett’s tests relative to mock infection.

## Results

### *Lactobacillus* strains vary in growth in various media types

**Fig 1:**
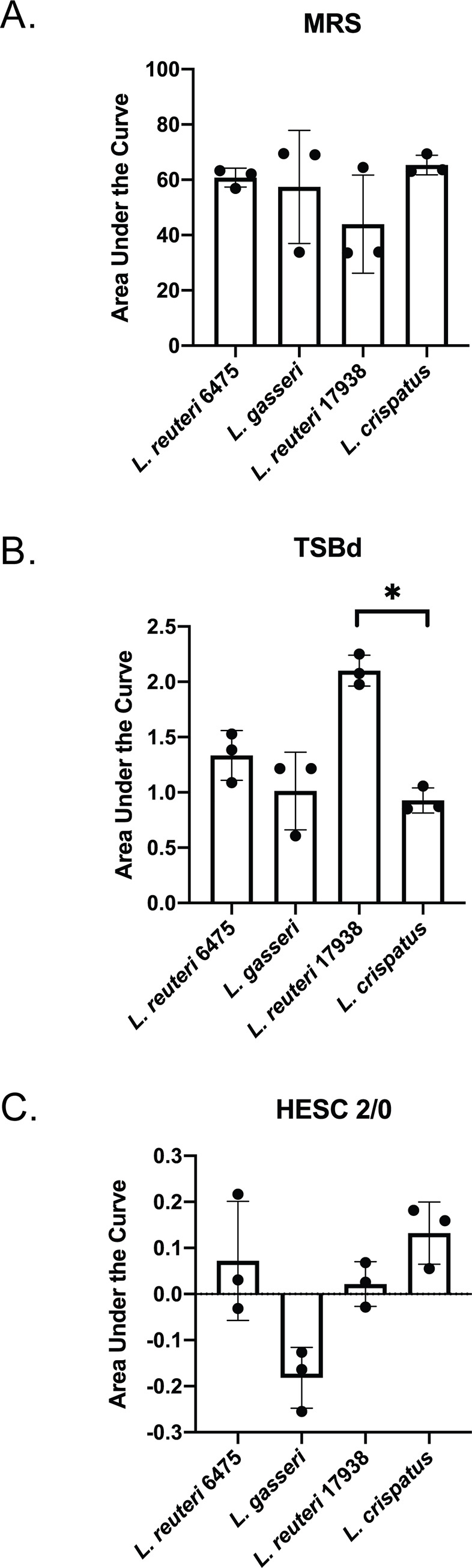
*Lactobacillus* growth varies by media type. *Lactobacillus* strains were grown overnight, washed in PBS, and inoculated into fresh: **A)** MRS media; **B)** TSA + 1% Dextrose; or **C)** HESC 2/0 media. The optical density 595 (OD_595_) was measured every 30 minutes for 48 hours using a spectrophotometer. For each graph, the Area Under the Curve (AUC) was calculated using GraphPad Prism 6. Differences were determined by comparing the AUC of three biological replicates by unpaired ANOVA (*p<0.05). Error bars represent the standard deviation between three independent biological experiments.

To determine growth phenotypes of four *Lactobacillus* strains in the various media types required for experiments, growth curves were performed. All strains grew in MRS and demonstrated similar lengths of lag phase and growth rates; however, they did differ in the length of exponential phase and maximum OD_595_. *Lr*6475 entered stationary phase earliest at 10 hours and reached a maximum OD_595_ of 1.5 at 12 hours. *Lg* 33323 reached a similar maximum OD_595_ of 1.5 but entered stationary phase three hours later, while *Lr*17938 reached the lowest OD_595_ of 1.14 after 14 hours of growth. *Lc*19390 reached the highest OD_595_ of 1.73 but also entered stationary phase later, which occurred after ~20 hours into growth. Overall, an evaluation of the Area Under the Curve (AUC) detected no significant differences in growth among all four strains in MRS (Fig 1A). TSB containing 1% dextrose (TSBd) has been used previously to evaluate biofilms in GBS [28]; therefore, we sought to characterize *Lactobacillus* biofilms in this media as well (Fig 1B). Each of the *Lactobacillus* strains grew poorly in the media. *Lc*19390 and *Lg*33323 grew similarly, each reaching a maximum OD_595_ of 0.02. *Lr*6475 grew slightly better, reaching an OD_595_ of 0.04, while *Lr*17938 grew significantly better than *L*. *crispatus*, reaching 0.06 in 48 hours. The worst growth conditions were observed for HESC minimal media containing 2% per volume fetal bovine serum and 0% antibiotics and antimycotics. None of the *Lactobacillus* strains grew in the cell culture media and none reached an OD_595_ above 0.005 (Fig 1C).

### *Lactobacillus* biofilm formation varies and is dependent on growth media

**Fig 2:**
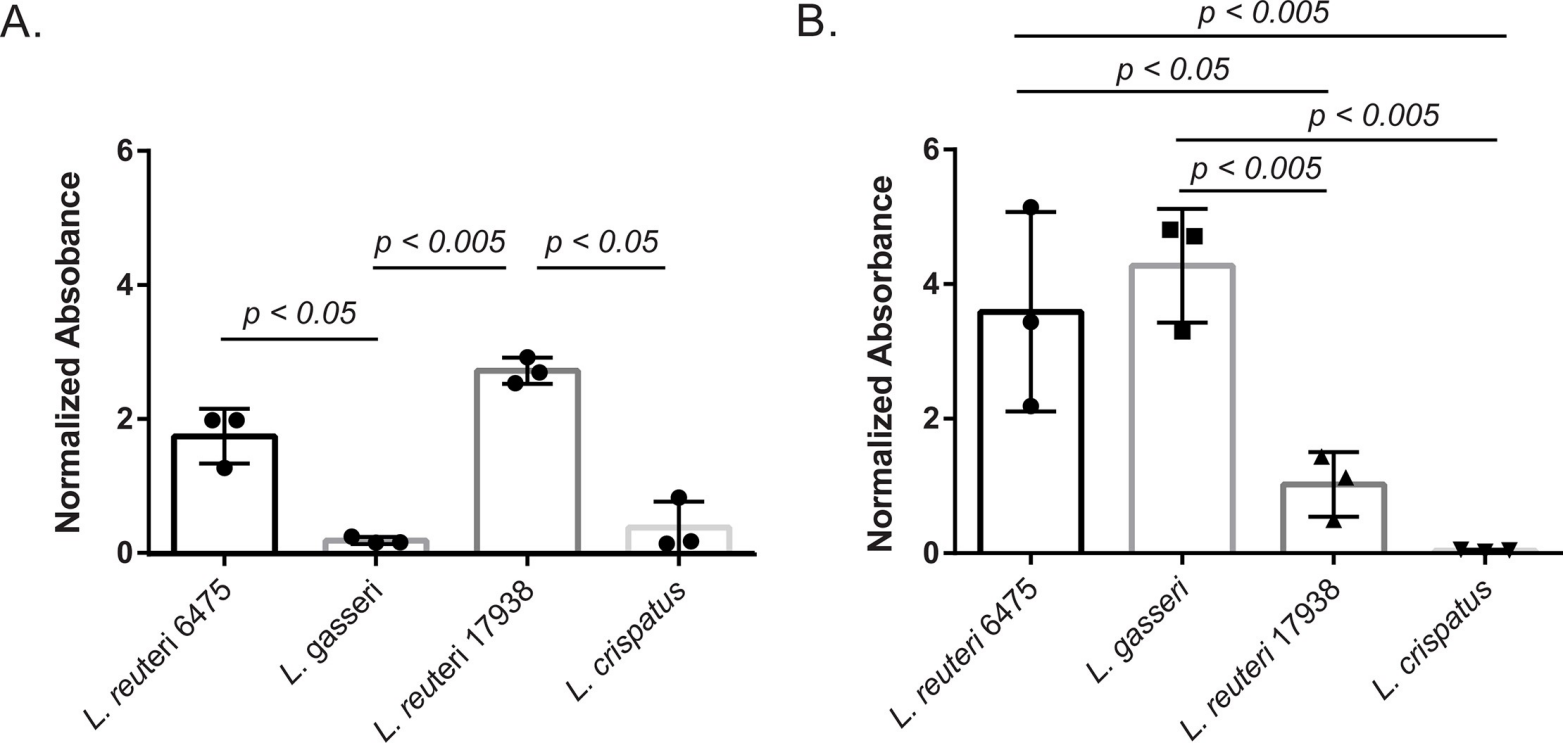
*Lactobacillus* species differ in the ability to form biofilms depending on media type. *Lactobacillus* was grown overnight, washed in PBS and incubated in: **A)** TSB with 5% dextrose; or **B)** MRS media for 48 hours in a 96-well plate. Normalized absorbance was calculated by subtracting the OD_595_ from the control well and multiplying by four to account for dilutions. Experiments were performed in biological triplicates of technical quadruplicates. Error bars represent standard deviation between biological trials. Significance was determined by an unpaired ANOVA (p<0.05) after confirmation of a normal distribution of the data via the Shapiro-Wilk test.

Bacterial biofilms have been shown to play a role in adherence to surfaces and persistence in hosts [24]; therefore, we evaluated biofilm production in all four *Lactobacillus* strains. Strong (OD_595_ = 1.13–7.00), weak (OD_595_ = 1.13–0.25), and non-biofilm (OD_595_ <0.25) production cutoffs are defined in the methods. Interestingly, biofilm formation was dependent on media type (Fig 2). *L*. *reuteri* 6475 and *L*. *gasseri* 33323 formed significantly better biofilms in MRS media (p < 0.05; p < 0.00005) compared to TSBd. Conversely, *L*. *reuteri* 17938 formed the strongest biofilm in TSBd compared to MRS (p < 0.05). While there was not a significant difference in biofilm formation for *L*. *crispatus* between media types, it did form a higher biofilm in TSBd, increasing from 0.039 (non-biofilm former) to 0.37 (weak biofilm former).

There were also differences between *Lactobacillus* strains within media types. In TSBd media, for instance, both *L*. *reuteri* strains formed stronger biofilms, reaching ODs of 1.74 and 2.71, respectively (Fig 2A). *L*. *crispatus* formed a weak biofilm of 0.37, whereas *L*. *gasseri* was classified as a non-biofilm former in TSBd, falling below the ODc at 0.18. In this media, biofilms formed by *L*. *reuteri* 17938 were significantly stronger than both *L*. *gasseri* and *L*. *crispatus* biofilms (p < 0.005, 0.005, respectively; unpaired ANOVA). *L*. *reuteri* 6475 was only significantly stronger than the *L*. *gasseri* biofilm (p < 0.05, unpaired ANOVA). There was no significant difference in biofilm production between *L*. *gasseri* and *L*. *crispatus* (p > 0.05).

In the MRS media (Fig 2B), biofilms formed by *L*. *reuteri* 6475 were significantly stronger than *L*. *reuteri* 17938 (p < 0.05) and *L*. *crispatus* (p< 0.005) but not *L*. *gasseri* (p > 0.05). Biofilms formed by *L*. *gasseri* were also significantly weaker than *L*. *reuteri* 17938 (p < 0.005) and *L*. *crispatus* (p< 0.005) in MRS media, though no difference was observed between *L*. *reuteri* 17938 and *L*. *crispatus* (p > 0.05).

### *L*. *crispatus* associates with decidual cells significantly better than other strains

**Fig 3:**
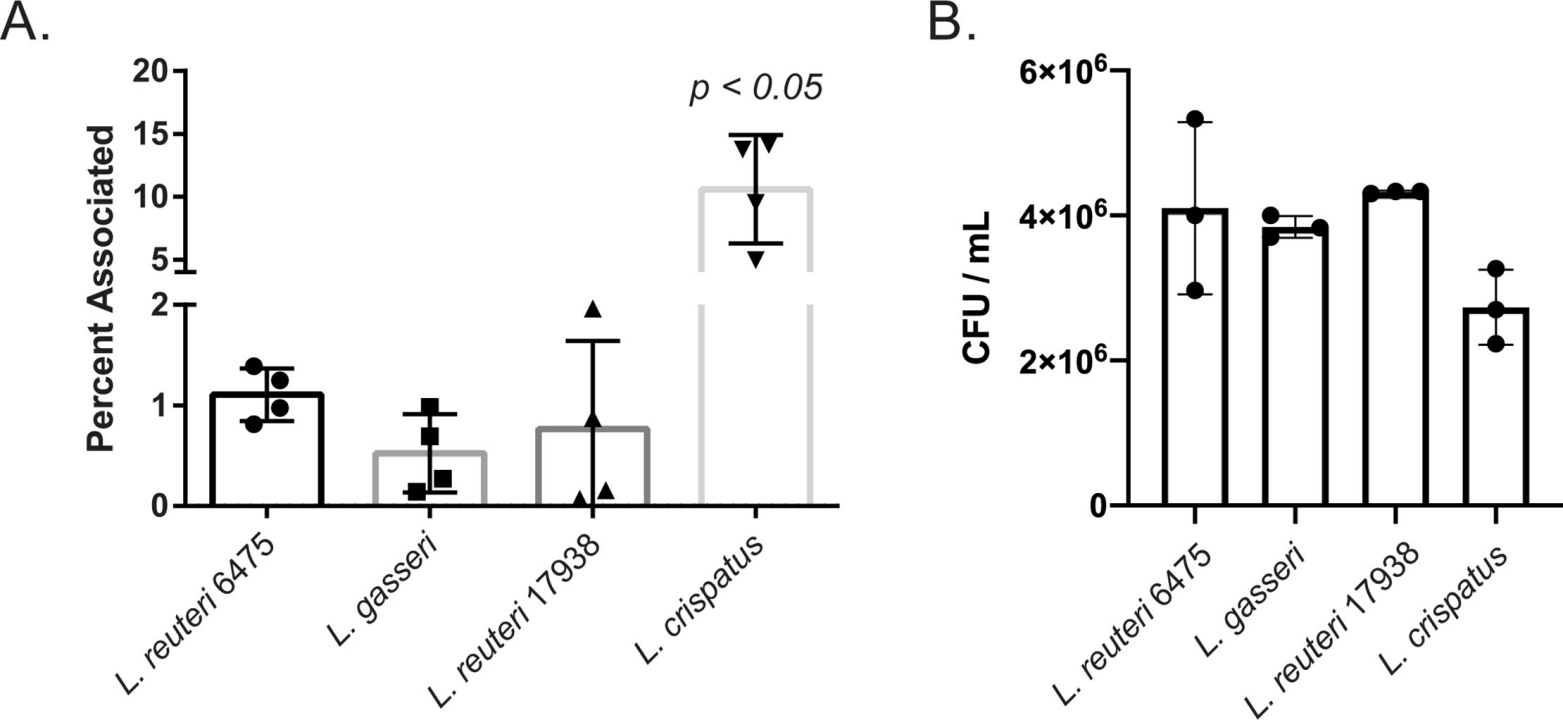
*Lactobacillus* associates with decidualized human endometrial stromal cells (dT-HESCs). **A**) The dT-HESCs were incubated with *Lactobacillus* at a MOI of 10 for two hours. The percent of associated bacteria was calculated relative to the total number of bacteria in the well. **B**) *Lactobacillus* were incubated for two hours in the presence of Triton-X and colony forming units (CFUs/ml) were quantified. Experiments were completed in biological quadruplets of technical triplicates. Error bars represent standard deviation between biological trials. Error bars represent standard deviation between biological trials, and significance differences were tested using an unpaired ANOVA (*p<0.05).

A key step to interaction with host cells is attachment. To determine if different *Lactobacillus* strains can interact with dT-HESCs, association assays were performed. As this is the first assay with *Lactobacillus* using this cell type, we compared association levels with those published for GBS, a pathogen known to colonize decidual cells. Indeed, we have previously used this experimental design to show that GBS association with dT-HESCs varied between 0.02 to 0.6% [20]. This level of association is similar to what we observed with lactobacilli, suggesting that *Lactobacillus* is also capable of associating with this cell type. Nonetheless, variation between strains was observed (Fig 3B). Of the strains tested, *L*. *crispatus* associated with host cells significantly better than the other strains with 10.6% of the total bacteria in the well establishing a stable association with the cells (p < 0.0005, unpaired ANOVA). The other strains had similar levels of association that were not significantly different from each other (p > 0.05); 1.11% of *L*. *reuteri* 6475, 0.52% of *L*. *gasseri* and 0.77% of *L*. *reuteri* 17938 associated with the dT-HESCs. To ensure that differences observed herein were not a result of differential survival in Triton-X, we confirmed equivalent survival in Triton-X across the four *Lactobacillus* strains (Fig 3A; p > 0.05).

### *Lactobacillus* does not induce phosphorylation of p38 in dT-HESCs

**Fig 4:**
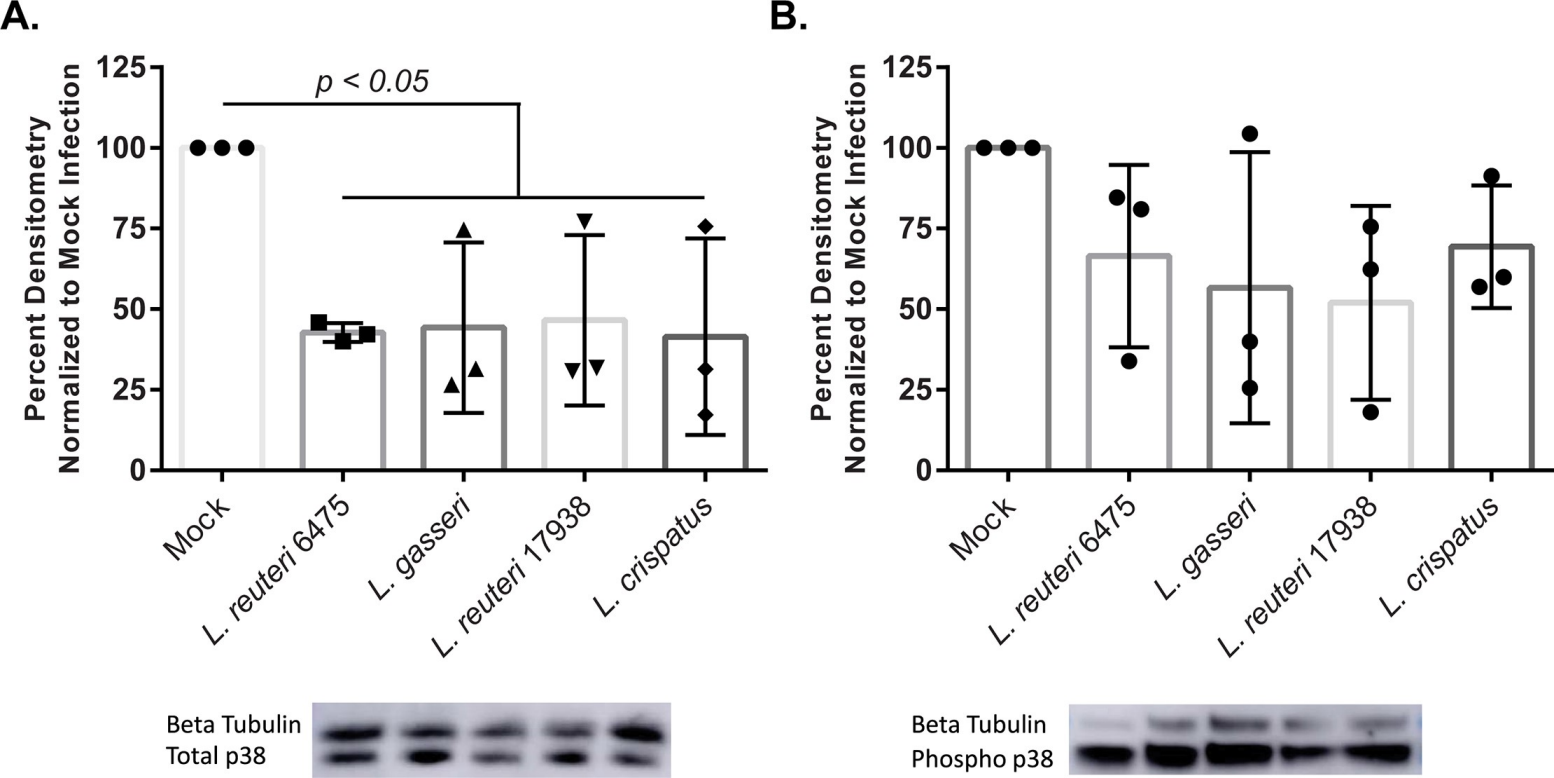
*Lactobacillus* affects total p38 and phosphorylated (Phospho) levels of p38. dT-HESCs were incubated with *Lactobacillus* at a MOI of 10 for three hours. Protein lysates were collected and analyzed via Western Blotting using densitometry in Image J to determine the amounts of **A**) total p38 and **B**) total Phospho p38. A β-tubulin internal loading control was used to account for loading differences by normalizing levels per well to the total protein levels. Graphed densitometry data is presented as a percent of the mock control for each of three biological replicates. Error bars represent the standard deviation of the data. Significance was determined using an unpaired ANOVA and a post-hoc Dunnett’s test.

As *Lactobacillus* associated with dT-HESCs, we next sought to assess if they triggered an immune response in this cell line. To do this, we evaluated levels of p38, a key player in the MAPK pathway that promotes cell death and inflammation [33]. Indeed, prior studies have found that *Lactobacillus* can affect levels of this protein and protect against invading pathogens [21, 34–37]. Western blots were used to quantify the level of the phosphorylated (active) form of p38 as well as total p38 after a three-hour infection with *Lactobacillus*. In comparison to a mock infection with cell culture media, each *Lactobacillus* strain lowered the levels of total p38, but did not affect the phosphorylated form of p38 (Fig 4A). Compared to the mock infection, total p38 was significantly reduced to 42.7%, 44.3%, 46.6% and 41.5%, respectively, for *L*. *reuteri* 6475, *L*. *gasseri*, *L*. *reuteri* 17938 and *L*. *crispatus* (p < 0.05). There were no significant differences between the *Lactobacillus* strains (p > 0.05). While levels of phosphorylated p38 were also decreased with the addition of *Lactobacillus* in comparison to the mock infection, these results were not statistically significant (p> 0.05). *L*. *reuteri* 6475, *L*. *gasseri*, *L*. *reuteri* 17938 and *L*. *crispatus* reduced levels of phosphorylated p38 to 66.5%, 56.7%, 52.0% and 69.4%, respectively (Fig 4B); no differences were observed between strains. The inconsistent level of β-tubulin across wells, however, could have prevented the detection of significant differences among strains given that protein values were normalized to the β-tubulin values. To further determine if *Lactobacillus* had any anti-inflammatory effects, we utilized an ELISA to detect changes in IL-10 secretion. No change in IL-10 production was observed as very low production of this cytokine was observed in both the mock and lactobacilli-treated cells.

### *Lactobacillus* does not trigger death in dT-HESCs

**Fig 5:**
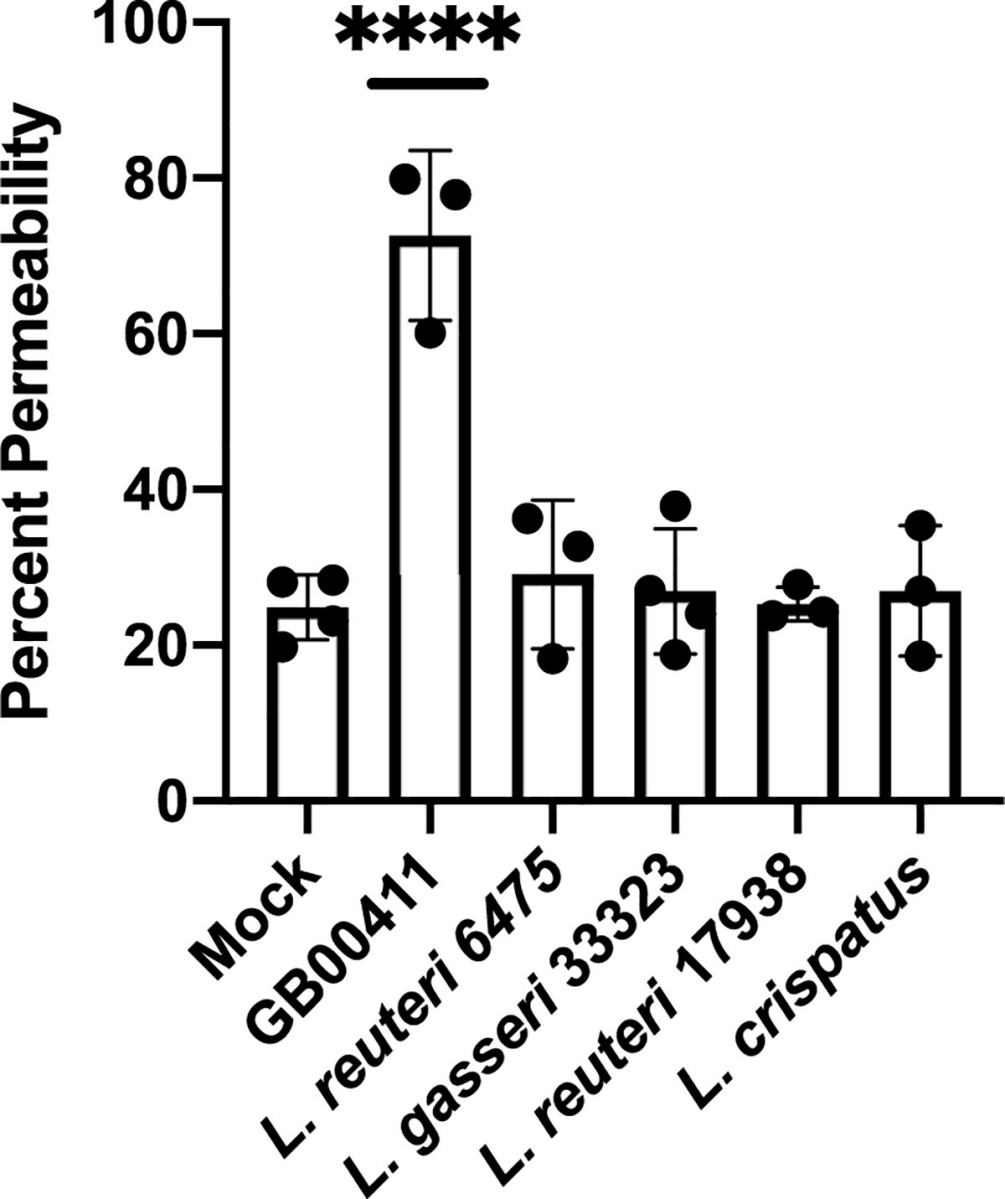
*Lactobacillus* does not induce dT-HESC death. dT-HESCs were incubated with *Lactobacillus* at a MOI of 10 and incubated for four hours. Cell permeability was detected using an ethidium homodimer assay, and percent permeability was calculated by lysing the remaining cell in each well. Group B streptococcal strain, GB00411, was included as a positive control. Graphed data represents three biological replicates, and the error bars represent the standard deviation of the data. Significance was determined using an unpaired ANOVA.

Reduction in total p38 and maintenance of the phosphorylated form led us to assess downstream effects of the MAPK pathway to determine the effect of decreased total p38 levels. Because this pathway is associated with cell death [33], we compared host cell permeability during incubation with *Lactobacillus* to that of a mock infection as a marker of host cell death (Fig 5). Group B streptococcal strain, GB00411, was previously shown to induce cell death [29] and was included as a positive control. No significant differences in the host cell permeability were observed with infection across the four *Lactobacillus* strains (p> 0.05, unpaired ANOVA). The media control contributed to a 24.9% cell death rate, while *L*. *reuteri* 6475, *L*. *gasseri*, *L*. *reuteri* 17938 and *L*. *crispatus* caused 29.1%, 26.93%, 30.2% and 27.0% cell death, respectively. No differences were observed between the individual strains (p> 0.05, unpaired ANOVA).

## Discussion

*Lactobacillus* has been studied as a probiotic in the gut and urogenital tract, but its effects in the placental membranes remain uncharacterized. As members of this bacterial species are being proposed as a probiotic for use during pregnancy [21, 38], it is important to understand the potential effects of *Lactobacillus* colonization of the placental membranes. Previous work has found vast differences in probiotic capacity between species of *Lactobacillus* as well as strains within the same species [39]; therefore, we first sought to characterize traits that may affect their ability to interact with host cell membranes including growth and biofilm formation. Growth differences were observed between strains and growth medias. As HESC 2/0 media lacks critical nutrients, it is not surprising that all four of the *Lactobacillus* strains were unable to grow. The inability to grow in this model system may affect some traits such as the production of secondary metabolites, which are important for survival in any system. Indeed, growth in anaerobic versus aerobic conditions, which may be more relevant for the placenta, should also be evaluated as our experiments were performed solely in the presence of 5% CO_2_ without altering the oxygen level.

To colonize the placental membranes, lactobacilli must be able to interact with the outermost layer of cells, the decidualized stromal cells. Indeed, we have demonstrated that each of the *Lactobacillus* strains could attach to dT-HESCs, though variation was observed in the percent association across strains. The overall level of association to dT-HESCs was similar to that described for GBS, an opportunistic pathogen that can attach to and invade the dT-HESCs [32, 40]. Notably, the *L*. *crispatus* strain was significantly better at associating with the decidualized stromal cells, which provides support for the identification of *L*. *crispatus* from the placental membranes of healthy, term pregnancies using metagenomics in a prior study [4]. Since these researchers also detected *L*. *acidophilus*, future work should examine whether it is also more capable of associating with decidual cells. The ability to attach to the placental membranes suggests that these strains may be able to serve as a barrier to invading pathogens as their presence may remove potential attachment sites for those bacteria. Further work coculturing *Lactobacillus* with different pathogens known to colonize the placental membranes should be completed to assess this potential.

The ability to form a biofilm has been associated with persistent colonization of many environments, and strains of *Lactobacillus* have been found to persist in other host sites including the intestines for up to a week [41]. We demonstrated that the strength of *Lactobacillus* biofilms observed in our assays were dependent on the type of growth media, highlighting the importance of nutrients in *Lactobacillus* biofilm production. Indeed, *L*. *reuteri* 6475 and *L*. *gasseri* 33323 formed significantly better biofilms in MRS media (p < 0.05; p < 0.00005), while *L*. *reuteri* 17938 formed a stronger biofilm in TSBd (p < 0.05). Differences in biofilm formation were also observed between individual *Lactobacillus* strains, though *L*. *gasseri* and *L*. *reuteri* 17938 were more drastically impacted by media type. These differences in biofilm production could impact a strain’s ability to persist and thrive in a given environment.

Premature birth and preterm premature rupture of the membranes (pPROM) are associated with inflammation and host cell death in the placental membranes [42]; therefore, it was important to determine whether *Lactobacillus* has an effect on immune signaling and host cell death. The MAPK pathway responds to stress and is involved in inflammatory signaling and cell death [33, 42]. The observation that total p38 levels were significantly reduced in all four strains suggests that this lower amount of total protein could reduce the overall output of the pathway, potentially stunting the ability of this pathway to promote host cell death. Though not significant, we also found a trend toward reduced levels of phosphorylated or active form of p38 in response to *Lactobacillus* compared to the mock infection, suggesting that *Lactobacillus* is not inducing this pathway. Although it has not been studied in dT-HESCs previously, *Lactobacillus* was shown to induce the MAPK pathway in macrophages, while other studies observed reduced MAPK induction in the gut, intestinal epithelial cells and the liver [21, 34–37]. Still, other work in a nematode model of *E*. *coli* sepsis showed that only certain strains of *Lactobacillus* further increased MAPK activation during infection, resulting in better survival outcomes [43]. Further work is therefore needed to more fully understand the effect of *Lactobacillus* on this pathway as well as its downstream effect on pregnancy outcomes. The reduced activation of the MAPK pathway combined with the lack of host cell death suggests that colonization by these *Lactobacillus* strain types would not likely result in damage to the placental membranes. As there are other cells types in the placental membranes, including macrophages, it would also be important to evaluate the effects of *Lactobacillus* in other cells and in a more complex model. In our experiments, there were no significant differences in host cell permeability after four hours of incubation. Using this same experimental design and conditions, the known pathogen, GBS, was found to cause ~70% host cell death [29] unlike the *Lactobacillus* strains tested herein (Fig 5). Together, these findings provide additional support for the finding that *Lactobacillus* fails to induce cell death in dT-HESCs *in vitro*.

In other systems, lactobacilli modulate the immune system to reduce inflammation or prime immune cells for invading pathogens [36]. Production of IL-10 is a marker of an anti-inflammatory response to *Lactobacillus*. This interleukin has previously been shown to be induced by *Lactobacillus* in other cell types including macrophage cell lines (RAW264.7 (mouse) and THP-1(human)), cervical tumor cells (HeLa), and colonic epithelial cells (Caco2) [36, 44, 45]. However, we observed little production of IL-10 in mock or following infection with all four strains of *Lactobacillus* in our study. Because it is possible that this cell line does not produce higher levels of cytokines in general, other models, such as whole decidua that produce higher cytokine levels, may serve as a better model of studying this interaction [22, 46].

Collectively, these data suggest that different strains of *Lactobacillus* sustain a commensal relationship with cells of the decidua. We observed variation among strains of *Lactobacillus* in growth, biofilm production and association with dT-HESCs, suggesting that this relationship will be strain-dependent, as suggested in the literature for other body sites. Further research needs to be done to examine if these characteristics allow *Lactobacillus* to perform the barrier function against pathogen invasion as was seen in the vaginal tract. These strains should also be studied in more complex models to examine the effect of the other cell types in the decidua, as they may alter the relationship. Since the placental membranes play such an important role in maintaining a healthy pregnancy, increasing our understanding of how traditional commensal bacteria affect these membranes could enhance knowledge of premature birth and other adverse pregnancy-related outcomes.
